# Wnt/β-catenin signaling promotes aging-associated hair graying in mice

**DOI:** 10.18632/oncotarget.20613

**Published:** 2017-09-01

**Authors:** Zhihui Zhang, Mingxing Lei, Haoran Xin, Chunyan Hu, Tian Yang, Yizhan Xing, Yuhong Li, Haiying Guo, Xiaohua Lian, Fang Deng

**Affiliations:** ^1^ Department of Cell Biology, Third Military Medical University, Chongqing, China; ^2^ Department of Cardiology, Southwest Hospital, Third Military Medical University, Chongqing, China; ^3^ Integrative Stem Cell Center, China Medical University Hospital, China Medical University, Taichung, Taiwan; ^4^ “111” Project Laboratory of Biomechanics and Tissue Repair & Key Laboratory of Biorheological Science and Technology of Ministry of Education, College of Bioengineering, Chongqing University, Chongqing, China; ^5^ Institute of New Drug Development, College of Biopharmaceutical and Food Sciences, China Medical University, Taichung, Taiwan; ^6^ Student Brigade Camp 3, Third Military Medical University, Chongqing, China

**Keywords:** Wnt/β-catenin, hair follicle, aging, melanocytes, hair graying, Gerotarget

## Abstract

Canities is an obvious sign of aging in mouse and human, shown as hair graying. Melanocytes in the hair follicle show cyclic activity with hair cycling, which transitions from anagen, catagen to telogen. How the hairs turn gray during aging is not completely uncovered. Here, by using immunostaining and LacZ staining in Dct-LacZ mice, we show that β-catenin is expressed in melanocytes during hair cycling. RT-PCR, western blot and immunostaining show that β-catenin expression is significantly increased in both anagen and telogen skin of aged mice, when compared to the anagen and telogen skin of young mice, respectively. Overexpression of Wnt10b not only accelerates hair follicle to enter anagen phase, but also promotes melanocytes differentiation in young adult mice (2-month old), with increased β-catenin expression in melanocytes at the secondary hair germ and matrix region of regenerated hair follicles. Overexpression of Wnt10b also promotes melanocyte progenitor cells differentiation *in vitro*. Our data suggest that increased Wnt signaling promotes excessive differentiation of melanocytes, leading to exhaustion of melanocyte stem cells and eventually canities in aged mice.

## INTRODUCTION

Aging is a physiological process associated with progressive structural and functional declines of tissues and organs [1]. The hair follicle is a mini-organ that undergoes repetitive cyclic regeneration under physiological conditions during postnatal life, thus supplying an excellent model for aging-associated disorders. Typical hair follicle aging phenotypes can be observed but not limited to several signs, such as irreversible hair loss, hair thinning and graying [2].

Regenerative hair cycling process in single hair follicle consists of three consecutive phases including growth phase (anagen), regression phase (catagen) and resting phase (telogen) [3-7]. Hair stem cell activation during telogen to anagen transition is mainly controlled by two reciprocal out of phases mechanisms. These include Wnt/β-catenin signaling pathway, which shows crucially roles in hair regeneration [8]. The other one is Bmp signaling pathway, which is decreased in competent telogen phase compared to the refractory telogen phase, leading to hair regeneration [9]. Melanocyte stem cells share the same niche with hair follicle stem cells. Progress has been making in unveiling regenerative behaviors and differentiation of melanocytes [10, 11]. Melanocyte stem cells are activated coordinately with hair follicle stem cells during hair regeneration. They migrate out from the bulge niche to the hair matrix region, and differentiate into melanocytes which generate melanin to pigment hairs.

There is increasing evidence showing that many morphogenetic pathways play key roles in regulating melanocytes behaviors. Of these, Wnt signaling functions as an important pathway controlling the patterning of melanocytes and influencing the decisions of melanocyte stem cells differentiation to pigment the hairs [12, 13]. Wnt3a induces melanocyte stem cell differentiation *in vivo* and *in vitro* [14]. Exogenous Wnt recruits β-catenin and Lef1 to bind the promoter of microphthalmia-associated transcription factor (MITF), which functions as a key gene that governs fates of melanocyte lineage cells [15]. Previous study shows that one of the visible signs of hair follicle aging is hair loss, which is due to decreased expression of Collagen XVII, causing the hair follicle stem cells differentiate into the epidermal lineages [2]. However, the mechanism of hair graying as the other obvious sign of hair follicle aging remains further investigation. Whether Wnt signaling acts as a positive or negative regulator in hair follicle aging is unclear.

Therefore, in this study, we first compared hair graying phenotype in young and adult mice. Since the important role of Wnt signaling in aging of other tissues, we examined periodic expression of β-catenin which is the effector of Wnt signaling pathway, in melanocyte lineage cells during hair cycling in Dct-Lac-Z CD1 transgenic mice. We also compared β-catenin expression at telogen phase and anagen phase skin in young mice and 34-month-old aged mice. To explore the function of Wnt signaling on melanocyte differentiation, we over expressed Wnt10b through adenovirus-mediated expression *in vivo* or *in vitro*, through intracutaneous injection of adenovirus into the young adult skin, or by adding them into iMC23 melanocyte stem cells, respectively. Our study indicates that Wnt signaling promotes differentiation of melanocyte stem cell, exhaustion of which leads to hair graying during aging.

## RESULTS

### Aging mice exhibit increased gray hairs

**Figure 1:**
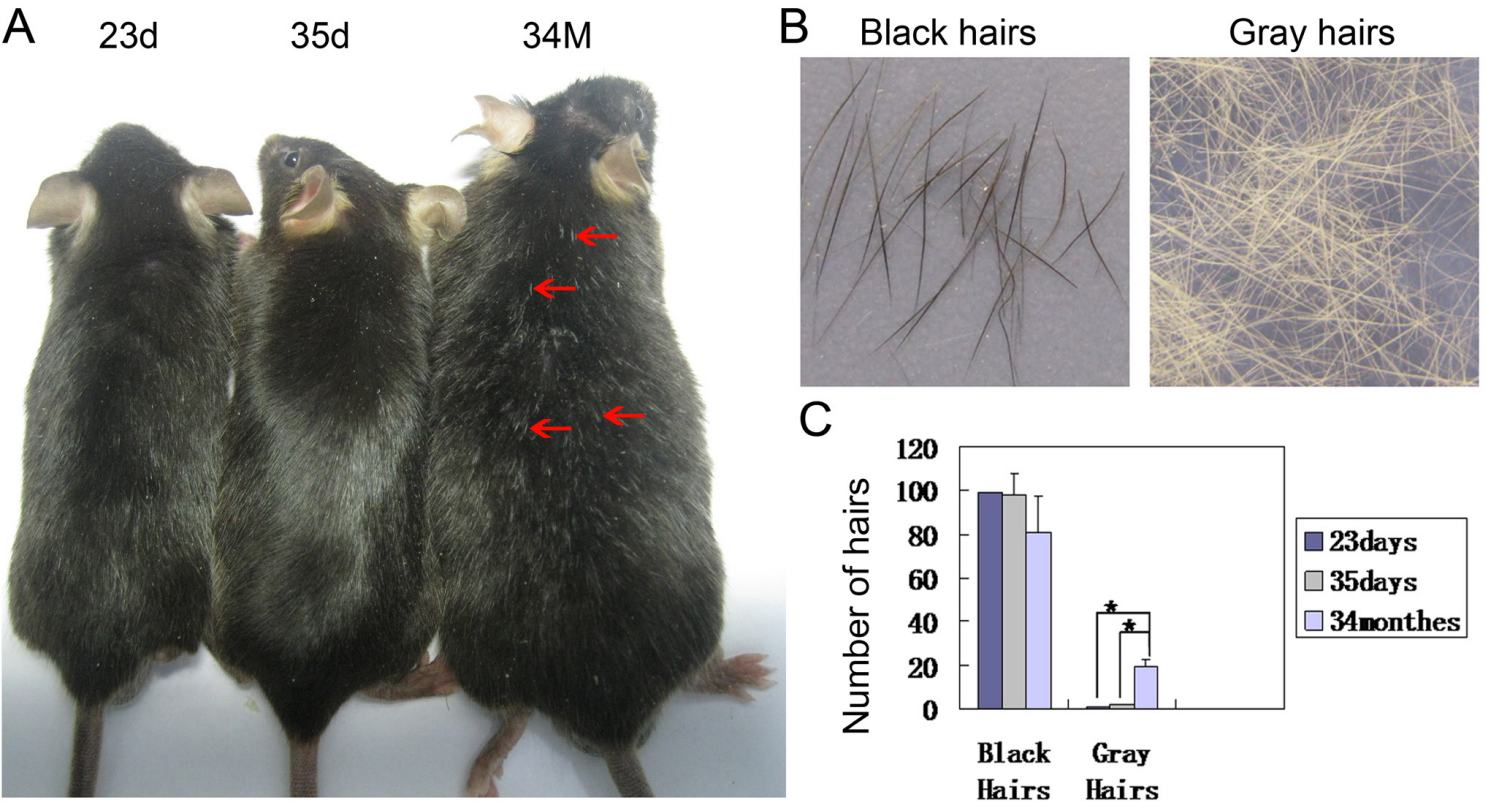
Hairs turn gray during aging in mice **A.** Partial hairs in the dorsal back skin turn gray in aged mice. **B.** Hairs plucked from young mice are black and old mice are gray. **C.** Statistical analysis reveals that significantly increased gray hairs in dorsal back skin in aged mice. 23d, postnatal 23 day; 34M, postnatal 34 months. *N* = 24.

To study the influence of aging on hair pigmentation in mice, we first counted and compared the number of black and gray hairs in dorsal back skin of C57Bl/6 mice aged at postnatal day 23 (23d, first telogen), 35d (second anagen) and 34 months (34M) (Figure 1A-1C). We observed that many hairs turn gray in mice aged at 34M, when compared to the young mice aged at 23d (Figure 1A). While the hairs in 23d young mice are black, the hair fiber completely turns gray from the distal part to the proximal part in 34M old mice (Figure 1B). Statistical analysis reveals that there are significantly more gray hairs in 34M old mice when compared to the 23d and 35d old mice (Figure 1C).

### β-catenin is expressed in melanocyte cell lineages

**Figure 2:**
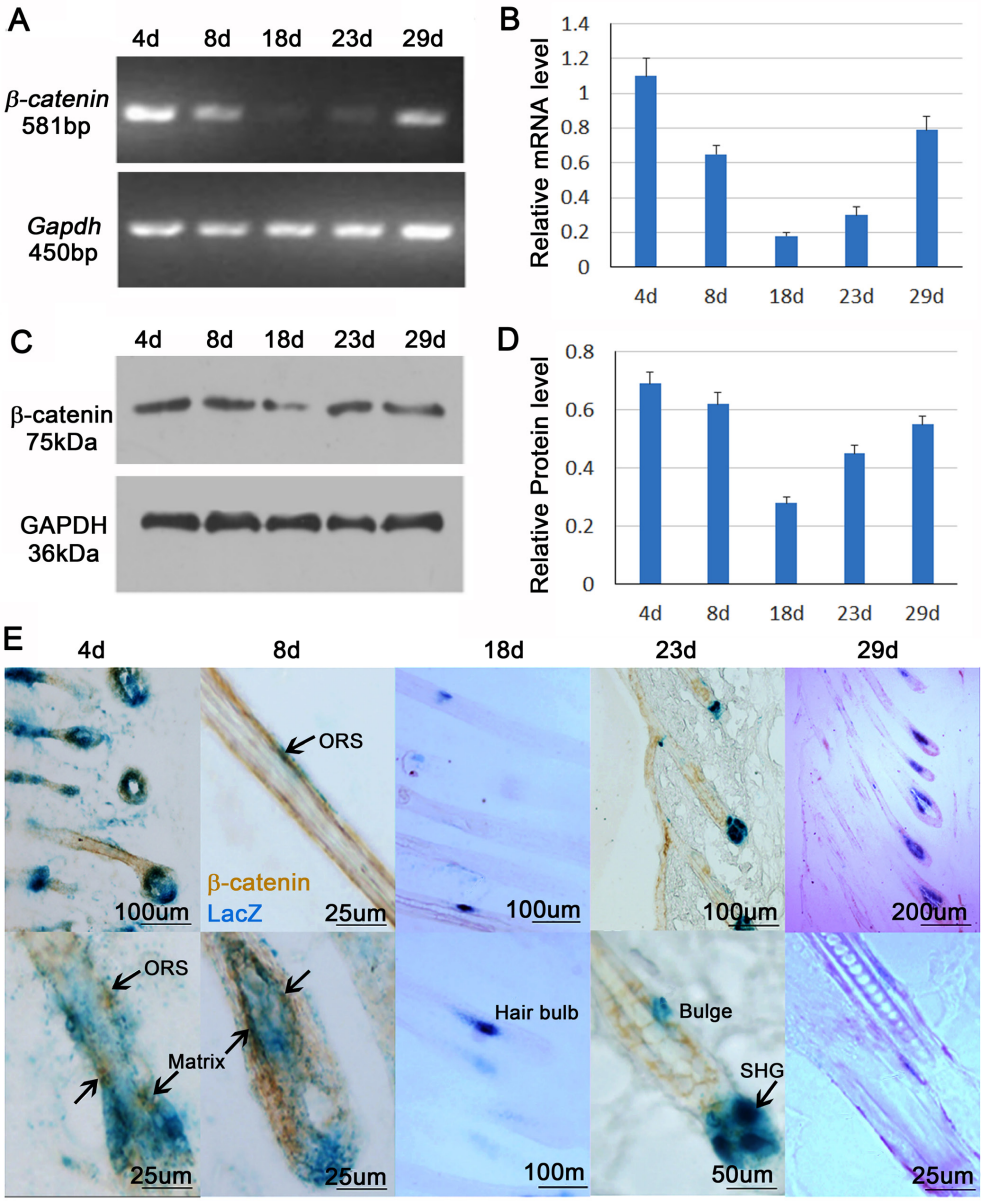
β-catenin expression in the melanocytes **A.** RT-PCR shows mRNA expression of β-catenin during hair cycling. **B.** Statistical analysis reveals that mRNA expression β-catenin is sig nificantly increased in anagen phase when compared to catagen and telogen phase. **C.** Western Blot shows protein expression of β-catenin during hair cycling. **D.** Statistical analysis reveals that protein expression of β-catenin is significantly increased in anagen phase when compared to catagen and telogen phase. **E.** β-catenin immunostaining and LacZ counterstaining show that nuclear β-catenin expression in melanocytes. ORS, outer root sheath; SHG, secondary hair germ. *N* = 12.

Wnt/β-catenin signaling is important for proliferation and differentiation of melanocytes. Previous study shows a cyclic expression pattern of β-catenin during hair cycling in C57BL/6 mouse strain [4]. Consistent with those studies, RT-PCR, western blot and their statistical analysis show that β-catenin is highly expressed in early and mid-anagen hair follicles (4d, 8d and 29d), but is decreased in catagen (18d) and telogen (23d) skin in Dct-LacZ transgenic CD1 mice (Figure 2A-2D).

To evaluate the expression of β-catenin in melanocyte cell lineages, we immunostained β-catenin expression in the skin of Dct-LacZ mouse, in which all melanocyte cell lineages are labeled by β-gal. This allows us to visualize LacZ+ melanocytes lineage cells, including melanoblasts, melanocyte stem cells, differentiating melanocytes, and mature melanocytes in the hair follicle. Translocation of β-catenin from cytoplasm to the nucleus indicates the activation of Wnt/β-catenin signaling pathway. We observed that nuclear β-catenin was only present in the early and mid-anagen phase of hair follicle. Close-up view reveals that nuclear β-catenin is expressed in the melanocytes located at hair matrix and secondary hair germ (Shg) region, and a few in the outer root sheath (Figure 2E). Combined with previous studies, our results further suggest that β-catenin regulates behaviors of melanocyte cell lineage during hair regeneration and growth.

### β-catenin expression is increased is skin of aged mice

**Figure 3:**
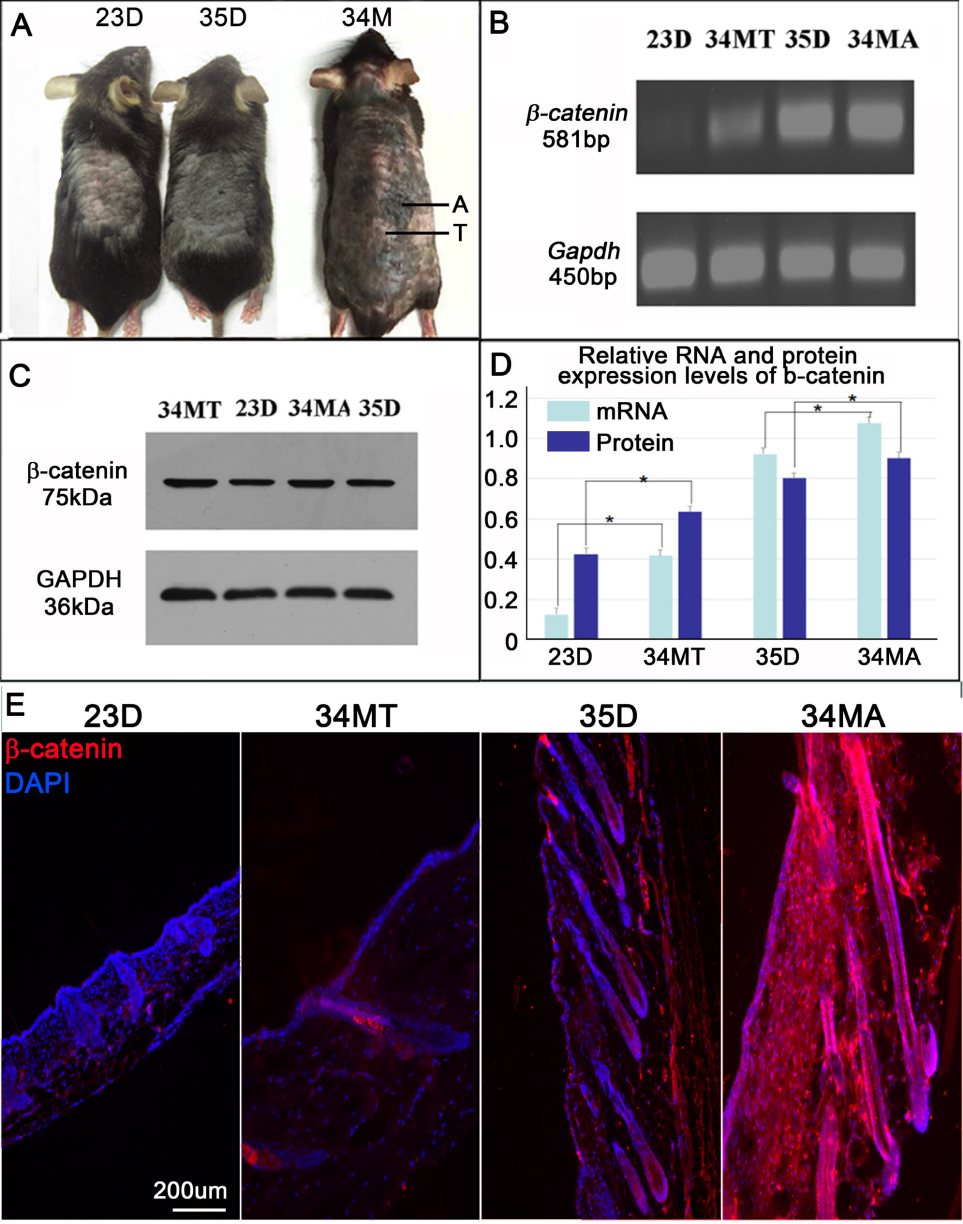
β-catenin expression is increased skin of aged mice **A.** Mice at different age. A, anagen; T, telogen. **B.** RT-PCR shows mRNA expression of β-catenin in mouse skin at different age. **C.** Western blot shows mRNA expression of β-catenin in mouse skin at different age. **D.** Statistical analysis reveals that mRNA and protein expressions of β-catenin are significantly increased in skin of aged mice, when compared to skin of young mice. **E.** Immunostaining shows β-catenin expression is highly expressed in anagen skin of aged mice. D, day; MT, month telogen; MA; month anagen. *N* = 12.

Several lines of evidence show that aging of some tissues and organs is related to the activation of Wnt/β-catenin signal pathway [16-18]. To explore the changes of β-catenin expression in skin during aging, we harvested dorsal back skin from both young and old Dct-LacZ CD1 mice (Figure 3A). We also compared skin samples at anagen or telogen phase of hair cycle by RT-PCR and western blot (Figure 3B-3C). Statistical analysis reveals that β-catenin expression was significantly increased both at 34 month telogen (34MT) skin and 34 month anagen (34MA) skin in aged mice, when compared to the young mice aged at 23d (telogen skin) and 35d (anagen skin), respectively (Figure 3D). Immunostaining further confirms that β-catenin expression is increased in both 34MT and 34MA skin when compared to the young mice skin aged at 23d and 35d, respectively. We observed that β-catenin expression is not only increased hair follicles of aged mice, but also increased at the dermal microenvironment.

### Overexpression of Wnt10b promotes anagen reentry with increased number of melanocytes in young mice *in vivo*

**Figure 4:**
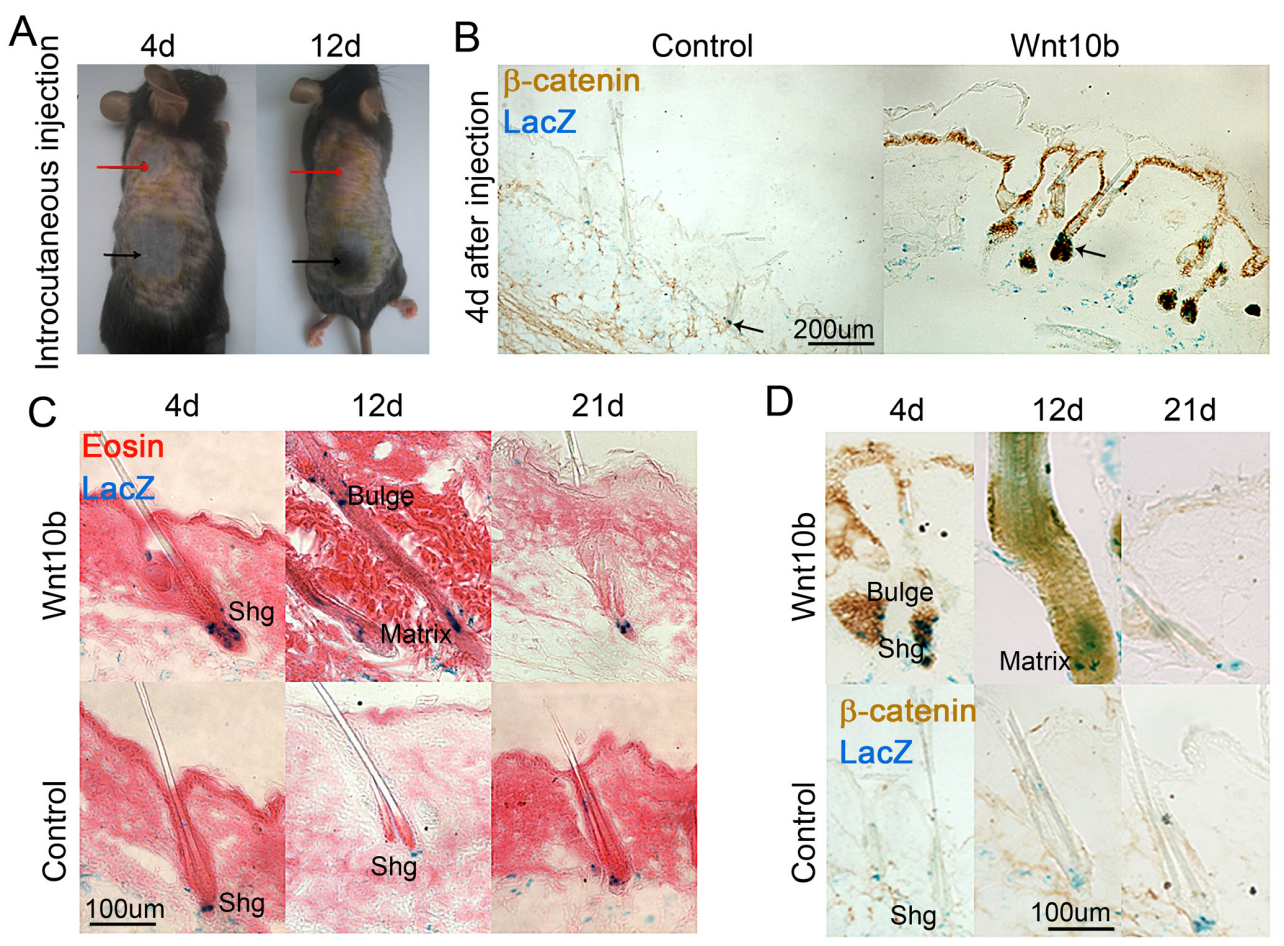
Overexpression of Wnt signaling promotes anagen reentry and melanocyte differentiation *in vivo* **A.** Intracutaneous injection of AdWnt10b results in accelerated hair regeneration in mice. **B.** Immunostaining and LacZ counterstaining show increased β-catenin expression in epidermis and hair follicles, and melanocytes differentiation in hair follicle and dermis, respectively, in AdWnt10b-overexpressed mice when compared to the AdGFP control group. **C.** Eosin and LacZ double staining shows that overexpression *Wnt10b* leads to increased melanocytes in secondary hair germ (Shg) region and hair matrix region. **D.** Immunostaining and LacZ counterstaining show nuclear β-catenin expression in melanocytes in AdWnt10b-overexpressed mice at secondary hair germ region and hair matrix, whereas the AdGFP control group shows no nuclear β-catenin expression. *N* = 12.

We previously showed that Wnt10b can induce hair regeneration [19]. Overexpression of Wnt10b also promotes more pigmentation of hair follicles [20]. To explore if Wnt signaling promotes melanocyte differentiation in hair follicles, we overexpressed *Wnt10b* by intradermal injection of adenovirus-mediated overexpression of *Wnt10b* (AdWnt10b) or control (AdGFP) into C57BL/6 mice at postnatal day 56 (P56), when the hair follicles are all at telogen phase. Consistent with previous studies done in C57BL/6 normal mouse [19], our result shows that hair follicles enter anagen phase represented by the black region where was injected with AdWnt10b, when compared to the control group which was injected with AdGFP and shows pink color that represents the telogen phase (Figure 4A). We also did this assay in Dct-LacZ mice, which allow us to better visualize melanocyte cell behavior after adenovirus treatment. Overexpression of *Wnt10b* leads to increased expression of β-catenin in skin epidermis and hair follicles, when compared to the AdGFP-treated control group, which shows little β-catenin expression (Figure 4B). β-gal staining reveals that the number of melanocyte lineage cells was largely increased in AdWnt10b-treated group, particularly in the hair follicle and dermis (Figure 4B). We further used β-gal staining counterstained with eosin to show melanocytes behavior during AdWnt10b-induced hair regeneration. The result shows that the number of melanocytes is increased at the secondary hair germ region of AdWnt10b-treated group, in which hair follicle starts to enter anagen phase (Figure 4C). The hair follicles progress to full anagen at 12 day and telogen at 21 days after AdWnt10b treatment. Whereas the hair follicles in AdGFP-treated group remain at telogen phase at corresponding time points paralleling to the AdWnt10b-treated group. When entering anagen phase, hair follicles treated with AdWnt10b show stronger and some nuclear β-catenin expression in melanocytes in the secondary hair germ and hair matrix region, when compared to the control group that remains at telogen phase with low expression of β-catenin (Figure 4D).

### Overexpression of Wnt10b promotes melanocyte progenitor cell differentiation *in vitro*

**Figure 5:**
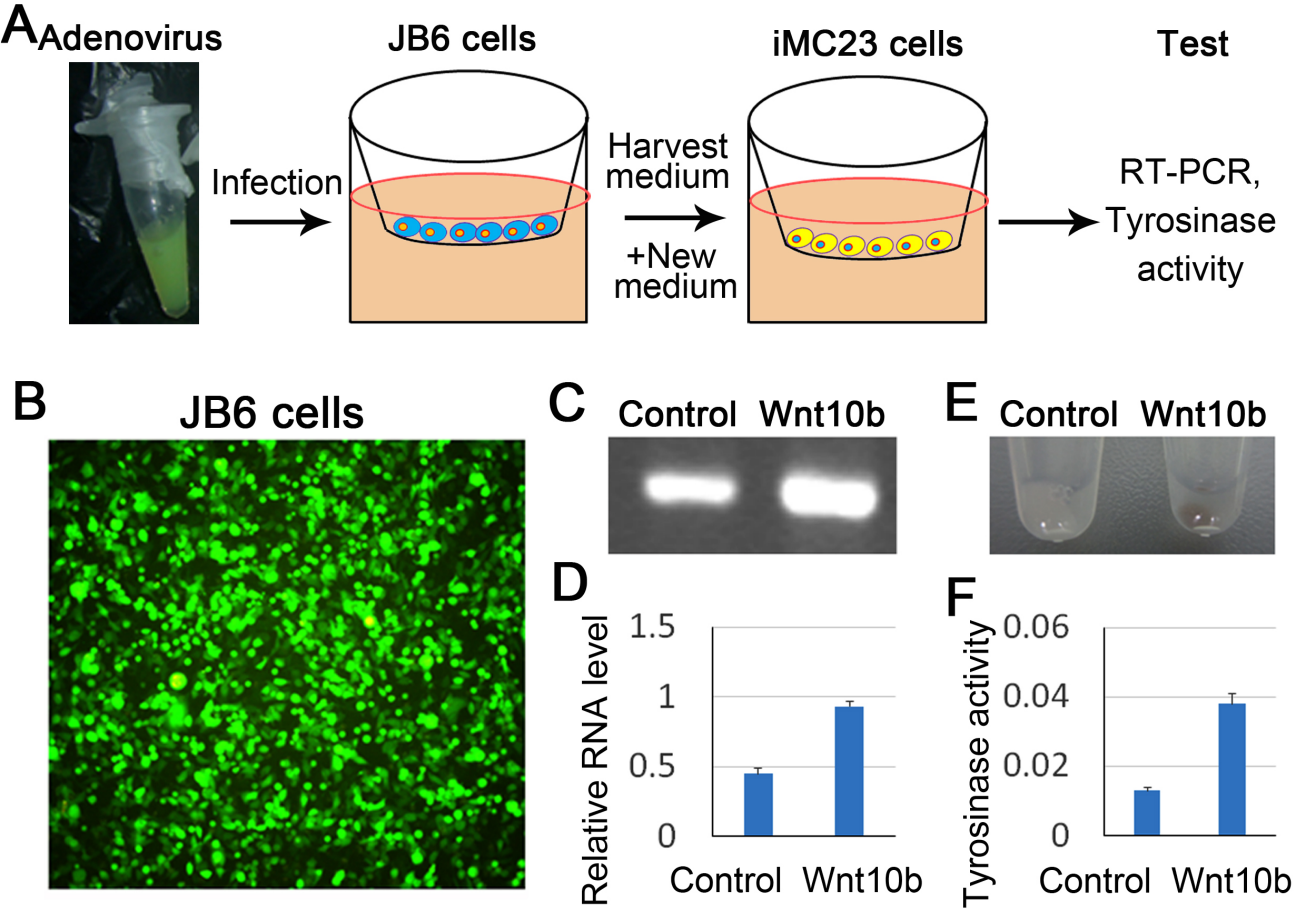
Overexpression of Wnt signaling promotes melanocyte stem cell differentiation *in vitro* **A.** Model of overexpression *Wnt10b* in iMC23 cells. **B.** Green fluorescence shows successful infection of adenovirus into JB6 cells. **C.** RT-PCR shows mRNA expression of β-catenin after Wnt10b treatment. **D.** Statistical analysis reveals that mRNA expression β-catenin is significantly increased in Wnt10b treated group compared to the control group. **E.** Melanogenesis after Wnt10b treatment. **F.** Tyrosinase activity assay of iMC23 cells was performed 2 days after the cells were treated by Wnt10b. *N* = 12.

To further confirm the role of Wnt/β-catenin in melanocyte differentiation, we treated melanocyte progenitor cells (iMC23 cells) with AdWnt10b *in vitro*. To reduce the toxicity of adenovirus on iMC23 cells, we first infected epidermal cells (JB6 cells) with AdWnt10b or AdGFP (control), and harvested the supernatant which was then added to the cultured iMC23 cells (Figure 5A). Robust green fluorescence produced by JB6 cells indicates successful infection of adenovirus to the cells (Figure 5B). RT-PCR and statistical analysis reveal that mRNA expression of β-catenin was significantly increased in iMC23 cells which are cultured by Wnt10b-containing medium for 12 hours, when compared to that of the control group (Figure 5C-5D). We also analyzed melanin production after the cells were cultured by Wnt10b-containing medium for 48 hours. The melanin production can be directly observed through the pigmented cell pellets after the iMC23 cells were digested and centrifugated to the bottom of the tube. The melanin formation was significantly increased in Wnt10b-treated iMC23 cells indicated by the black pellet, when compared to the control group in which showing gentle melanogenesis (Figure 5E). Moreover, the tyrosinase activity assay reflecting melanogenesis was used to test the influence of Wnt signaling on melanocyte differentiation. Tyrosinase activity is significantly increased in Wnt10b-treated group compared to the control group (Figure 5F). These data further suggest that Wnt10b/β-catenin signaling promotes melanocyte differentiation.

## DISCUSSION

Tissue aging is characterized by decline of regenerative ability. Previous studies show that loss of function or incomplete maintenance of stem cells lead to compromised tissue regeneration during aging [21]. Hair follicle stands at an idea model to study age-associated tissue regeneration, due to its cyclic behavior in postnatal lifetime [22-25]. Canities is an obvious sign of aging in mammals such as mouse and human, resulting in progressive hair graying during their aging process. Fundamental mechanisms controlling hair graying involve multiple possibilities. One is melanocyte stem cells are exhausted because of high activators that promote melanocyte stem cell differentiation [21]. The other is melanocyte stem cells sharing the same niche with hair follicle stem cells at bulge become silenced due to increased expression of inhibitors during aging process [26]. Though there may be other possibilities such as senescence-caused loss of function or terminal differentiation of stem cells [2].

**Figure 6:**
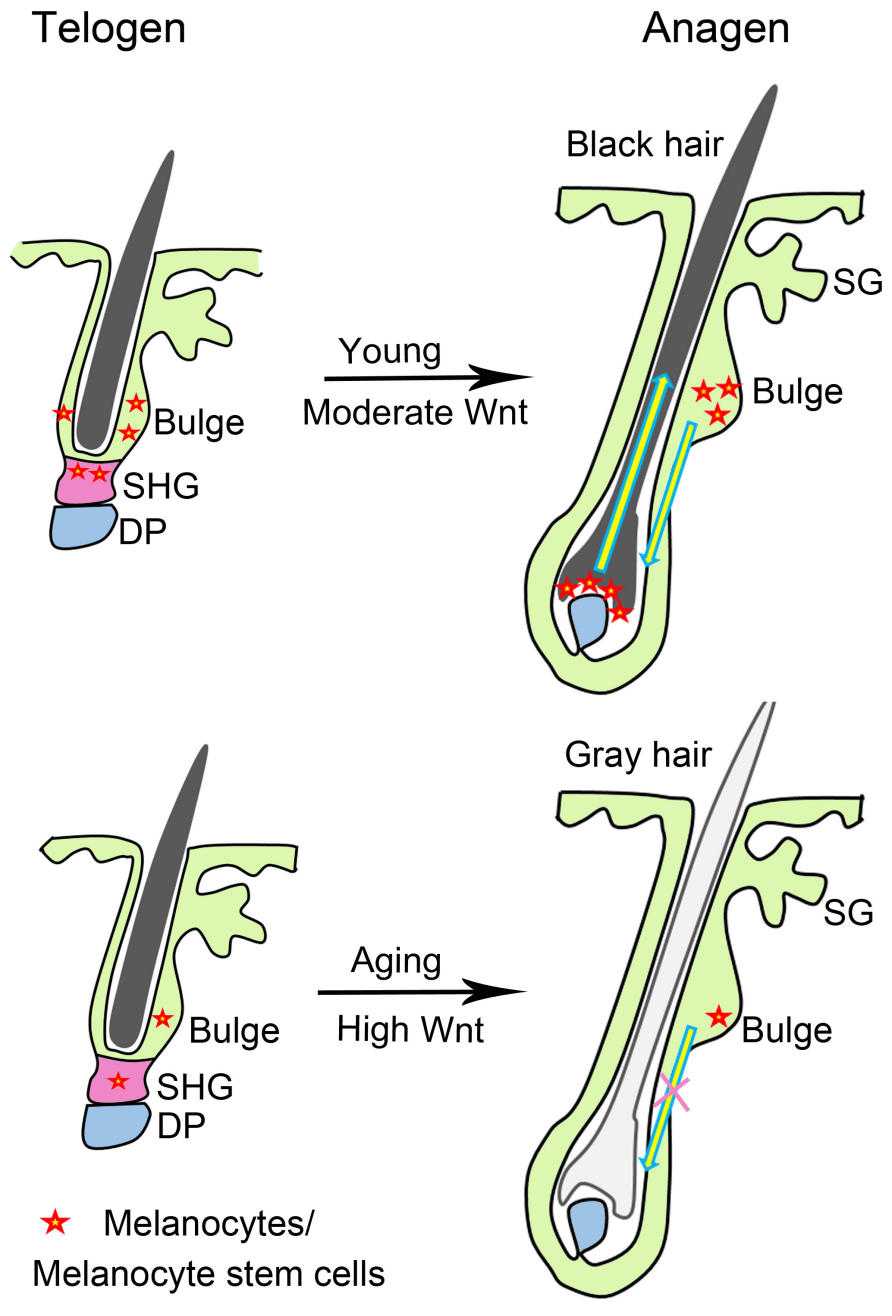
Schematic of Wnt signaling and hair graying Moderate Wnt signaling allows anagen reentry during hair regeneration, and promotes differentiation of melanocytes in hair follicle of young mice. High Wnt signaling promotes excessive differentiation of melanocytes, leading to decreased melanocyte stem cells which give rise to progressive hair graying during aging. DP, dermal papilla; SG, sebaceous gland; SHG, secondary hair germ.

Here, we found that β-catenin as the effector of Wnt signaling pathway was significantly increased in both telogen and anagen of aged mice skin, when compared to telogen and anagen of young mice skin, respectively. Particularly, β-catenin was expressed at the nucleus of some melanocytes during hair regeneration and growth, indicating the activation of Wnt signaling in these cells. Consistent with previous studies [12, 13], our study provide evidence that Wnt signaling promotes melanocytes differentiation. Moreover, our findings suggest that increased β-catenin expression in skin of adult mice may result in exhaustion of melanocyte stem cells in hair follicles, leading to hair graying during aging process (Figure 6).

Previous studies show that aging of tissues and organs is related to the classical Wnt/β-catenin signal pathway. However, it seems that Wnt signaling plays dual roles in contributing to aging among different tissues. Several studies demonstrate that Wnt signaling promotes aging of cells or tissues [16-18]. For example, increased Wnt signaling in klotho mice leads to decreased number of stem cell population and increased number of aged cells in the skin and small intestine, resulting in altered muscle stem cell fate and increased fibrosis [16]. Wnt signaling induces aging of mesenchymal stem cells through regulating p53/p21 pathway [18]. Whereas other studies show that Wnt signaling ameliorates aging of intestinal stem cells [27], neuronal tissue and skeletal muscle [28].

Wnt signaling usually maintains at a low level which is under the threshold for hair follicle stem cell activation at telogen [29]. Overexpression of β-catenin in epidermis induces ectopic hair follicle regeneration [30], and even tumorigenesis [31]. In our study, we found β-catenin expression is increased in telogen skin of aged mice compared to those in young mice. Increased expression of β-catenin should shorten the telogen phase, meaning an accelerated telogen to anagen reentry of the hair follicles. However, previous study shows that the length of telogen phase is gradually increased in duration during aging in mice, and this is because of decreased activators such as Follistatin and increased inhibitors such as Bmp2, Dkk1 and Sfrp4 that are periodically expressed by the extra-follicular macro-environment [26]. With increasing inhibitors expressed in skin during aging process, activators can also be induced through the reaction-diffusion (activator-inhibitor) mechanism. Thus, we suppose that the increased level of β-catenin in telogen hair follicle, particularly in the melanocytes stem cells or progenitor cells may contribute to melanocyte stem cell differentiation in the bulge niche, resulting in decreased number of melanocyte stem cells. Whereas the expression level of β-catenin is insufficient to counterbalance the increased inhibitors, leading to the inactivation of hair follicle stem cells and a longer telogen phase in the aged skin. In addition, different from telogen-anagen transition which is a collective behavior in the skin, hair graying in both mice and human tends to be an individual behavior, with gray hairs regeneration in a progressive process. We speculate that there is a differential expression level of β-catenin and its inhibitors in the bulge region of each hair follicle in the aged skin, leading to the progressive hair graying during aging in animal.

Indeed, previous study suggests that Wnt/ β-catenin signaling has distinct roles of in determine melanocyte stem cell fate [12]. Low level of Wnt/ β-catenin signaling is required to maintain the undifferentiated state of melanocyte stem cells, whereas high Wnt/ β-catenin signaling promotes melanocytes differentiation. This is controlled by regulation between β-catenin, Pax3 and MITF [32]. Hence, in anagen hair follicle of aged skin, increased Wnt/β-catenin signaling further promotes melanocytes terminal differentiation, leading to this lineage be exhausted. Our study further supports this hypothesis. Overexpression of *Wnt10b* not only accelerates hair follicles to enter anagen phase, but also promotes differentiation of melanocytes *in vivo*. Moreover, overexpression of *Wnt10b* in iMC23 cells promotes melanocyte stem cell differentiation, as indicated by melanogenesis and tyrosinase activity assays. Nevertheless, the exact β-catenin expression level that balances hair stem cell activation and melanocyte stem cell differentiation remains further investigation. Future study will evaluate if decrease of Wnt signaling can alleviate exhaustion of melanocyte stem cells in aged mice.

Since Wnt/β-catenin is also expressed in other regions of hair follicles where melanocytes were absent. For example, Wnt/β-catenin is expressed in the other regions of hair bulb as well as in the DP where melanocytes are absent. How does Wnt/β-catenin signaling coordinate the cellular behavior of other epithelial cell differentiation and melanocytes differentiation in the hair follicle? First, different regions express different Wnt receptors, which can sense different Wnt ligand to regulate cellular behaviors such as proliferation and differentiation. Second, Wnt signaling is also regulated by other signals which have differential expression in different regions of hair follicle; Third, epithelial-mesenchymal interaction also functions as a critical mechanism in regulating cellular behaviors in the hair follicle [33]. Mesenchymal cells may constitute micro-niches with different epithelial cell lineages, resulting in different epithelial-mesenchymal interactions [34].

In summary, our results demonstrate that β-catenin expression is increased in hair follicles undergoing aging, compared to the hair follicle in young mice at the corresponding cycle stages. Increased Wnt signaling is insufficient to induce hair regeneration but may promote melanocyte stem cell differentiation in hair follicle bulge niche, leading to the exhaustion of melanocyte stem cells during aging process (Figure 6). Our study provides a potential mechanism to modulate expression levels of activator or inhibitor in the skin microenvironment and macroenvironment, aiding in the development of a strategy for the treatment aging-associated degenerative disorders, such as canities.

## MATERIALS AND METHODS

### Mice and skin samples

Dct-LacZ CD1 transgenic mice were gifted from Professor Lan Jackson at the MRC Laboratory (UK). Animals were maintained in a SPF room at the Experimental Animal Center of the Third Military Medical University. For examining the expression of β-catenin, the dorsal back skins were harvested from the male mice at postnatal 4d (P4, early anagen of the first hair cycle), 8d (P8, mid-anagen of the first hair cycle), 18d (P18, catagen of the first hair cycle), 23d (P23, telogen of the first hair cycle) and 29d (P29, early anagen of the second hair cycle). Intracutaneous injection of adenovirus was performed as previously described [3, 19].

### Cell culture and tyrosinase activity assay

JB6 cells (ATCC, USA) were cultured in DMEM culture medium with 10% fetal bovine serum, in an atmosphere containing 5% CO2 in air at 37°C. Adenoviruses were generated and the supernatant of adenovirus-infected cells was harvested as previously described [35]. Then the supernatant was added to the iMC23 cells [36], which were cultured under the same condition as JB6 cells. For tyrosinase activity assay, iMC23 cells were treated with 1% TritonX-100/PBS for 30min and centrifuged. 50 μL supernatant was added with 10μL 2mg/ml L-DOPA and incubated at 37°C for 2 hours. The sample was measured at 480nm for absorbance.

### Reverse transcription and polymerase chain reaction (RT-PCR)

Total RNA was extracted from the skin specimens per the instruction for use of Trizol reagent. RT-PCR was performed per the instruction for use of the reverse transcription kit (TOYOBO, Japan) and PCR MIX kit (Tiangen, China). The primers for β-catenin were designed as: 5’-ATCACTGAGCCTGCCATCTG-3’ (sense); 5’-GTTGCCACGCCTTCATTCC -3’ (antisense), with a 581bp product in length. The annealing temperature was set at 63°C.

### Western-blot

Proteins were extracted from the skin specimens by using of RIPA lysis buffer (Beyontime, China). The extracts were vortexed, centrifuged at 12000×g for 10min. The concentration was determined by using a BCA protein concentration determination kit (Beyontime, China). Then the extracts were added with the loading buffer and incubated in boiling water for 5 min, followed by electrophoresis (concentrating gel 80V, 30 min; separating gel 100V, 90 min). The proteins were transferred to a PVDF membrane (250 mA, 2 h), which was treated with methanol for 1 min and electrotransfer buffer for 15 min before use. After wash with TBST (5min × 5), the PVDF membrane was blocked with 5% defatted milk (2 h), incubated with rabbit anti-β-catenin polyclonal antibody (1:1000; Abcam, USA) at 4°C for overnight, and secondary antibody (1:10000; Boster, China) at 37°C for 1 h, then developed with chemoluminescence.

### β-gal staining and immunohistochemistry

The back skins from Dct-Lac-Z CD1 transgenic mice were fixed at 4°C in 4% paraformaldehyde for 1 h, and stained with x-gal (Beyotime, China) for 24 h. After wash, the skin specimens were embedded in paraffin and sliced into 5 μm sections, which were incubated with rabbit anti-β-catenin polyclonal antibody (1:600; Abcam, USA) at 4℃ for overnight, and the secondary antibody (Zhongshan, China) at 37°C for 1h. The samples were developed with DAB (Zhongshan, China).

### Data acquisition and statistical analysis

RT-PCR and Western blot images were analyzed with Quantity One software to acquire the optical density of the bands. The relative expression level was normalized at the ratio of optical density of target band against that of internal control band. All experiments in this study were repeated at least three times. Data were expressed as x̄ ±S and One-way ANOVA of data using SPSS10.0 software.
